# Association Between Polycystic Ovary Syndrome and Markers of Subclinical Atherosclerosis in Premenopausal Women: A Systematic Review

**DOI:** 10.3390/jcm15135197

**Published:** 2026-07-02

**Authors:** Eman Elsheikh, Khalil Ibrahim Bograin, Nourah Mashari Alqadri, Maryam Khalid Alquaymi, Shahad Ahmad Alhikan, Shahad Adel Balghonaim, Amjad Salah Alsaleem, Dalal Saad Alsulaiman, Raneem Khalid Alateeq, Sadeem Khalid Almulhim, Hala Mohammed Alqahtani, Maryam Mohammed Al Dhaif

**Affiliations:** 1 Internal Medicine Department, College of Medicine, King Faisal University, Al-Ahsa 36362, Saudi Arabia; 2 Cardiology Department, Faculty of Medicine, Tanta University Hospital, Tanta 31111, Egypt; 3 Alahas PHC Center, Alahsa 35353, Saudi Arabia; 4 College of Medicine, King Faisal University, Al-Ahsa 36362, Saudi Arabia

**Keywords:** polycystic ovary syndrome, subclinical atherosclerosis, carotid intima-media thickness, cardiovascular risk, premenopausal women, PCOS, endothelial dysfunction

## Abstract

**Background/Objectives**: Polycystic ovary syndrome (PCOS) is increasingly recognized as a condition associated with cardiometabolic risk, yet its relationship with subclinical atherosclerosis in premenopausal women remains unclear. This systematic review aimed to synthesize and critically appraise the available evidence on the association between PCOS and markers of subclinical atherosclerosis. **Methods**: A systematic search of PubMed/MEDLINE, Embase, Scopus, Web of Science, and the Cochrane Library was conducted for studies published between January 2021 and January 2026. Eligible studies included premenopausal women with PCOS and assessed direct vascular markers or validated surrogate indicators of subclinical atherosclerosis. Data were synthesized narratively following PRISMA guidelines. **Results**: Nine studies were included, comprising five human clinical studies and four bioinformatics analyses. Evidence from imaging-based studies demonstrated increased carotid intima-media thickness, reduced wall shear stress, and a higher prevalence of subclinical vascular abnormalities in women with PCOS, particularly in some hyperandrogenic phenotypes. In contrast, adolescent populations showed predominantly metabolic and inflammatory alterations without clear structural vascular changes. Biochemical studies reported adverse lipid profiles and elevated atherogenic markers, while mechanistic studies highlighted inflammatory and mitochondrial pathways. **Conclusions**: Current evidence suggests that PCOS may be associated with early vascular alterations in premenopausal women; however, findings are limited by heterogeneity and observational designs. Further large-scale, phenotype-stratified prospective studies using standardized vascular assessments are needed to clarify cardiovascular risk in this population.

## 1. Introduction

Polycystic ovary syndrome (PCOS) is the most common endocrine disorder affecting women of reproductive age, with reported prevalence estimates ranging from 5% to 18% depending on the diagnostic criteria applied and the populations studied [1]. In clinical practice, PCOS is primarily defined by combinations of hyperandrogenism, ovulatory dysfunction, and polycystic ovarian morphology, most commonly according to the Rotterdam criteria [2]. Although traditionally approached as a reproductive and gynecological disorder, PCOS is now increasingly recognized as a complex multisystem condition with important metabolic and cardiovascular implications [3]. Women with PCOS frequently exhibit insulin resistance, central adiposity, dyslipidemia, impaired glucose regulation, and elevated blood pressure, all of which are well-established contributors to long-term cardiovascular risk [4].

Interest in the cardiovascular consequences of PCOS has grown substantially over the past two decades. Beyond the clustering of conventional cardiometabolic risk factors, several studies suggest that women with PCOS may also demonstrate early vascular abnormalities before the onset of overt cardiovascular disease [5]. In particular, markers of subclinical atherosclerosis—such as carotid intima-media thickness (CIMT), carotid intima thickness, altered wall shear stress, and other vascular imaging parameters—have attracted increasing attention as potentially sensitive indicators of early arterial injury in young and middle-aged women with PCOS [6]. These measures are especially relevant in premenopausal populations, in whom clinically manifest cardiovascular disease is still relatively uncommon, but early pathological vascular changes may already be present [7].

The biological plausibility of an association between PCOS and early atherosclerotic change is strong. Insulin resistance and compensatory hyperinsulinemia may amplify androgen production and worsen metabolic dysfunction, while chronic low-grade inflammation, oxidative stress, endothelial dysfunction, and adverse lipid profiles may together promote vascular remodeling and arterial injury [8]. However, plausible mechanisms alone do not establish the extent or consistency of clinical vascular involvement in women with PCOS [9]. The key clinical question is not simply whether PCOS is linked to cardiometabolic abnormalities, but whether premenopausal women with PCOS demonstrate measurable evidence of subclinical atherosclerosis when compared with women without PCOS [10].

Despite growing literature in this field, the evidence remains difficult to interpret for several reasons. First, studies vary considerably in the diagnostic criteria used for PCOS, including Rotterdam, National Institutes of Health, and Androgen Excess Society definitions, which capture phenotypes with different metabolic and androgenic profiles [11]. Second, the vascular outcomes assessed across studies are heterogeneous, ranging from direct imaging-based measures of subclinical atherosclerosis to indirect biochemical surrogates and broader cardiovascular risk indicators [12]. Third, many studies are cross-sectional, include relatively small samples, and differ in the degree to which they account for major confounders such as obesity, insulin resistance, and hypertension. As a result, the literature has often been interpreted in ways that blur the distinction between direct evidence of vascular change and indirect evidence of cardiometabolic risk.

These challenges are particularly important in relation to previous reviews and narrative discussions of PCOS and cardiovascular disease, which have sometimes combined highly heterogeneous forms of evidence under a single broad framing of “atherosclerosis.” Such an approach risks overstating the strength and coherence of the evidence base [13,14,15,16]. A more clinically meaningful synthesis requires clearer prioritization of direct human evidence of subclinical vascular change, while distinguishing this from surrogate biochemical markers or mechanistic hypotheses. In this context, premenopausal women represent the most appropriate population for focused review, because this stage of life allows assessment of early vascular changes before the confounding influence of menopausal transition and age-related cardiovascular disease becomes more prominent [17].

Accordingly, this systematic review aimed to synthesize and critically appraise the available human clinical evidence on the association between polycystic ovary syndrome (PCOS) and markers of subclinical atherosclerosis in premenopausal women [18]. To enhance clinical interpretability, the review prioritized direct human vascular assessments, particularly imaging-based outcomes such as carotid intima-media thickness and related vascular parameters, while considering validated biochemical surrogate markers as supportive indirect evidence. Exploratory bioinformatics and mechanistic studies were evaluated separately and interpreted as hypothesis-generating evidence rather than direct clinical evidence [19]. By adopting this structured approach, the review sought to provide a balanced and clinically relevant assessment of early vascular alterations associated with PCOS, identify important gaps in the current literature, and inform future research on cardiovascular risk stratification in this population.

## 2. Materials and Methods

### 2.1. Study Design and Reporting Framework

This systematic review was conducted and reported in accordance with the Preferred Reporting Items for Systematic Reviews and Meta-Analyses (PRISMA) 2020 statement. The objective was to synthesize and critically appraise the available human clinical evidence on the association between polycystic ovary syndrome (PCOS) and markers of subclinical atherosclerosis in premenopausal women. Although a formal prospective registration was not performed, the review methodology was predefined and followed a structured protocol to ensure transparency and reproducibility.

### 2.2. Review Question and PICOS Framework

The review question was formulated according to the PICOS framework (Table 1). The population comprised premenopausal women with a confirmed diagnosis of PCOS. The exposure was the presence of PCOS, irrespective of disease duration, treatment status, or phenotypic subtype. The comparator consisted of women without PCOS. The primary outcomes were direct vascular markers of subclinical atherosclerosis, including carotid intima-media thickness (CIMT), carotid intima thickness, wall shear stress, arterial stiffness parameters, and other imaging- or vascular function-based measures. Secondary outcomes included validated biochemical surrogate markers of atherosclerotic risk, such as the atherogenic index of plasma and related endothelial or lipid-associated biomarkers. Study designs eligible for inclusion were human observational studies, including cross-sectional, case–control, and cohort studies. This framing was intentionally adopted to prioritize clinically interpretable human evidence and to avoid conflating direct vascular findings with exploratory mechanistic or bioinformatic data.

### 2.3. Eligibility Criteria

Studies were considered eligible if they met all of the following criteria: (1) included premenopausal women or reproductive-age women with a confirmed diagnosis of PCOS based on recognized diagnostic criteria, such as the Rotterdam, National Institutes of Health (NIH), or Androgen Excess Society (AES) criteria; (2) included a comparator group of women without PCOS; (3) evaluated at least one direct vascular marker of subclinical atherosclerosis or a validated biochemical surrogate relevant to early atherosclerotic risk; (4) used an observational design, including cross-sectional, case–control, or cohort methodology; and (5) reported original data in sufficient detail to permit extraction of study characteristics and findings. Studies were excluded if they included postmenopausal women without separately extractable premenopausal data, relied on self-reported PCOS without diagnostic confirmation, focused exclusively on conventional cardiovascular risk factors without any vascular or validated surrogate atherosclerotic outcome, lacked a non-PCOS comparator group, were case reports, editorials, narrative reviews, conference abstracts without adequate full-text data, or were non-human studies. To improve internal coherence, exploratory bioinformatics and transcriptomic studies were not treated as equivalent to clinical studies in the principal evidence synthesis; where retained, they were considered separately as hypothesis-generating evidence only.

### 2.4. Information Sources and Search Strategy

A comprehensive literature search was undertaken across the following electronic databases: PubMed/MEDLINE, Embase, Scopus, Web of Science, and the Cochrane Library. The search period covered studies published from January 2021 to January 2026, in line with the original review scope and the intention to capture recent evidence generated under contemporary diagnostic and vascular assessment approaches. The search strategy combined controlled vocabulary terms and free-text keywords related to PCOS and subclinical atherosclerosis. Core PCOS terms included “polycystic ovary syndrome,” “PCOS,” “polycystic ovarian syndrome,” and related endocrine terminology. Core outcome terms included “atherosclerosis,” “carotid intima-media thickness,” “CIMT,” “endothelial dysfunction,” “arterial stiffness,” “flow-mediated dilation,” and related vascular terms. Boolean operators were used to maximize sensitivity and specificity. Manual screening of reference lists from included studies and relevant review articles was also performed to identify additional potentially eligible studies. The full database-specific search strategies are presented in Table 2.

### 2.5. Study Selection

All retrieved records were exported to Rayyan for deduplication and screening. Duplicate records were removed using Rayyan’s automated detection function followed by manual verification. Study selection was performed in two stages. First, two reviewers independently screened titles and abstracts against the predefined eligibility criteria. Second, the full texts of potentially eligible studies were independently reviewed by the same two reviewers. Disagreements at either stage were resolved through discussion and, when necessary, consultation with a third reviewer. Reasons for exclusion at the full-text stage were documented and are presented in the PRISMA flow diagram and, where possible, in a supplementary exclusions table. Because the original review process identified a substantial number of records for which full text could not be accessed, the handling of inaccessible reports requires explicit transparency. Institutional access routes, reference-chain searching, and author contact were used where feasible before exclusion of inaccessible reports.

Because 32 of the 50 full-text reports sought for retrieval could not be accessed, the completeness of the evidence base may have been affected by selection bias. These inaccessible reports may have included eligible studies with findings that differed from those included in the final synthesis. Therefore, the results of this review should be interpreted with caution, and the possibility that the available evidence does not fully represent the broader literature should be acknowledged.

### 2.6. Data Extraction

A standardized data extraction form was developed in Microsoft Excel and pilot-tested before full extraction. Two reviewers independently extracted data from each included study to enhance accuracy and reduce extraction errors. Disagreements were resolved through discussion, with third-reviewer arbitration if necessary. Extracted information included: first author, year of publication, country, study design, sample size, participant characteristics, diagnostic criteria used for PCOS, comparator characteristics, age and body mass index data, key inclusion and exclusion criteria, vascular or biochemical outcomes assessed, measurement methods, and principal findings. For imaging studies, details of the vascular assessment protocol, including CIMT or ultrasound-based measures, were extracted whenever available. For studies reporting surrogate biochemical markers, the specific biomarker, method of measurement, and direction of association were recorded. Potential confounding variables, including obesity, insulin resistance, androgen excess, lipid abnormalities, and blood pressure, were also documented where reported. This extraction strategy was adapted from the original manuscript and proposal but refined to better distinguish direct vascular evidence from indirect surrogate findings.

### 2.7. Risk of Bias and Methodological Quality Assessment

Methodological quality was assessed independently by two reviewers using design-specific appraisal tools. Cross-sectional studies were appraised using the Joanna Briggs Institute Critical Appraisal Checklist for Analytical Cross-Sectional Studies, while case–control and cohort studies were evaluated using the Newcastle–Ottawa Scale or design-appropriate adaptations. The assessment focused on participant selection, validity of PCOS diagnosis, appropriateness of comparison groups, reliability of outcome measurement, control of confounding, and adequacy of statistical analysis. Risk-of-bias findings were incorporated into the interpretation of the results rather than presented as isolated methodological information. Studies with direct vascular imaging outcomes and stronger control of bias were given greater interpretive weight, while cross-sectional studies, small samples, and studies relying mainly on surrogate biochemical markers were interpreted more cautiously. Bioinformatics studies were not included in the formal clinical risk-of-bias assessment because they did not directly address the primary clinical review question and were considered exploratory mechanistic evidence only.

### 2.8. Certainty of Evidence

The certainty of evidence was considered using GRADE principles, including risk of bias, inconsistency, indirectness, imprecision, and publication bias. Overall, the certainty of evidence was judged to be low to very low. This judgment reflects the predominance of observational designs, modest sample sizes, heterogeneity in PCOS diagnostic criteria and phenotype distribution, variation in vascular and biochemical outcome definitions, limited confounder adjustment in several studies, and the large number of inaccessible full-text reports. The certainty was relatively stronger for direct imaging-based evidence showing increased carotid intima-media thickness and related vascular abnormalities in adult women with PCOS, but remained limited because only a small number of studies contributed to these findings. Evidence from biochemical and bioinformatics studies was judged to be indirect and hypothesis-generating rather than confirmatory.

### 2.9. Data Synthesis

Given the expected heterogeneity in study designs, participant characteristics, PCOS phenotypes, vascular assessment methods, and reported outcomes, a narrative synthesis was performed as the primary method of evidence integration. Studies were grouped according to outcome type to preserve conceptual clarity. First, studies evaluating direct vascular imaging or vascular function markers were synthesized as the principal clinical evidence base. Second, studies assessing validated biochemical surrogate markers were synthesized separately as indirect evidence of atherosclerotic risk. Where exploratory mechanistic or transcriptomic studies were retained for contextual purposes, these were not integrated into the main clinical synthesis and were described separately as hypothesis-generating evidence. Within each group, studies were compared in terms of design, population characteristics, PCOS diagnostic criteria, outcome definitions, adjustment for confounders, and direction and consistency of findings. A quantitative meta-analysis was not performed unless studies were judged to be sufficiently homogeneous in design, outcome measurement, and reporting. This conservative synthesis approach was adopted to avoid over-integration of evidence types with different clinical interpretability and to align the review more closely with a rigorous systematic review framework.

### 2.10. Handling of Missing Data and Inaccessible Reports

When study reports lacked key methodological or outcome details, available Supplementary Materials were reviewed to obtain missing information. If essential data remained unclear, the information was recorded as not reported. For studies identified during screening but unavailable in full text, exclusion was not treated as a routine procedural step; instead, such cases were documented explicitly because inaccessible reports may introduce selection bias and affect the completeness of the evidence base.

No ethical approval was required for this study as it is a systematic review based on previously published data.

## 3. Results

### 3.1. Study Selection and PRISMA Flow

**Figure 1:**
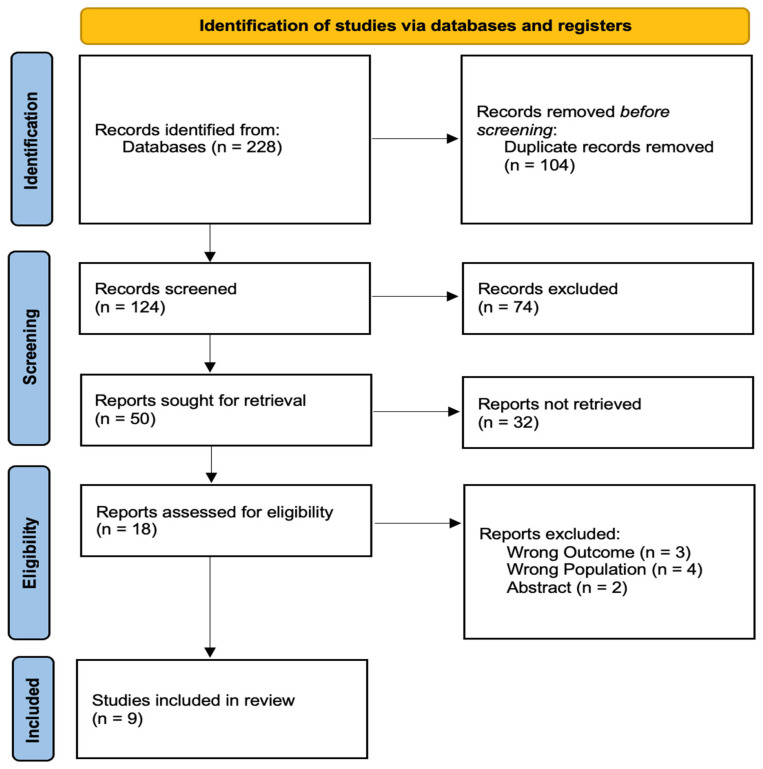
PRISMA 2020 flow diagram of study identification, screening, eligibility assessment, and inclusion.

The database search identified 228 records. After removal of 104 duplicates, 124 unique records remained for title and abstract screening. At this stage, 74 records were excluded because they did not meet the predefined eligibility criteria. Fifty full-text articles were sought for retrieval. Of these, 32 reports could not be retrieved because full-text access was unavailable (Figure 1). The remaining 18 full-text articles were assessed for eligibility, of which 9 were excluded for the following reasons: wrong outcome (*n* = 3), wrong population (*n* = 4), and conference abstract without sufficient data (*n* = 2). Ultimately, 9 studies were included in the review. These comprised 5 human clinical studies, which formed the main evidence base for the review question, and 4 bioinformatics studies, which were retained only as exploratory mechanistic evidence and were not considered equivalent to the clinical findings.

Because a substantial proportion of potentially eligible reports (32/50) could not be retrieved in full text, this should be interpreted as a possible source of selection bias. In keeping with the reviewer’s concern, this limitation should be acknowledged explicitly when interpreting the completeness and certainty of the evidence base.

### 3.2. Risk of Bias Assessment

The methodological quality of the included human clinical studies was assessed using appraisal principles appropriate to each observational design. Particular attention was given to the clarity of participant selection, validity of PCOS diagnosis, appropriateness of comparison groups, reliability of vascular or biochemical outcome assessment, identification and control of confounding factors, and adequacy of statistical analysis. Because the primary review question concerned human clinical evidence of subclinical atherosclerosis in premenopausal women with PCOS, the four bioinformatics and machine-learning studies were not incorporated into the formal clinical risk-of-bias assessment. Instead, they were retained as exploratory mechanistic evidence and interpreted separately from the main clinical synthesis (Table 3).

Overall, the methodological quality of the clinical evidence ranged from moderate to relatively strong. The two prospective controlled imaging studies by Dong et al. [22] and İncesu Çintesun et al. [21] were judged to have the lowest overall risk of bias among the included clinical studies. Both used clearly defined PCOS populations, appropriate comparison groups, and direct vascular outcome assessment with standardized imaging procedures. In Dong et al. [22], the use of ultra-high-frequency ultrasound and vector flow imaging strengthened measurement validity, although the modest sample size and residual confounding by body mass index, insulin resistance, and hyperandrogenism remain important considerations. In İncesu Çintesun et al., the inclusion of phenotype-based subgrouping and age-matched controls enhanced interpretability, but the single-center design and limited information on adjustment for all potential confounders prevented a judgment of unequivocally low risk across all domains [21].

The remaining three clinical studies were judged to have moderate risk of bias. Garoufi et al. [20] benefited from clear inclusion and exclusion criteria and objective measurement of cardiometabolic markers and cIMT, but the study was limited by a small adolescent sample, cross-sectional design, and reduced ability to account for confounding. In addition, the adolescent population may limit direct generalizability to the broader premenopausal population. Bykowska-Derda et al. [23] also showed moderate risk of bias because it was a cross-sectional study without a non-PCOS comparison group and relied on AIP as an indirect surrogate of atherosclerotic risk rather than direct vascular imaging. Dietary exposure was assessed using questionnaire-based self-report, which introduces the possibility of recall and reporting bias. Fadhil et al. [24] used a case-control design with age-matched controls and objective laboratory measurements, but risk of bias remained moderate because the study was single-center, relied on surrogate biochemical markers rather than direct vascular imaging, and provided limited adjustment for potentially important confounders such as adiposity, insulin resistance, and other metabolic factors.

### 3.3. Main Results

A total of nine studies met the inclusion criteria and were synthesized in this review. Of these, five human clinical studies formed the principal evidence base for the review question [17,18,19,20,21,22,23,24,25]. In contrast, four bioinformatics and machine-learning studies were retained as exploratory mechanistic evidence and interpreted separately. Overall, the findings were most consistent for an association between PCOS and early vascular abnormalities when assessed using direct imaging methods in adult women, while biochemical, dietary, and transcriptomic studies provided indirect or hypothesis-generating support for an adverse atherogenic profile in this population (Table 4).

### 3.4. Direct Vascular Imaging Evidence

The principal clinical evidence base consisted of five human studies, including two prospective controlled studies, two cross-sectional studies, and one case-control study, conducted in Greece, Turkey, China, Poland, and Iraq. Across these studies, PCOS was diagnosed predominantly using the Rotterdam criteria, and the assessed outcomes ranged from direct vascular imaging markers of subclinical atherosclerosis to indirect biochemical and dietary surrogate markers [20,21,22,23,24].

The strongest direct evidence of early vascular injury came from the two prospective controlled imaging studies. In the study by Dong et al. [22], women with PCOS had significantly greater carotid intima thickness and intima-media thickness than controls, together with significantly lower wall shear stress in the common carotid artery and carotid bulb. Importantly, subclinical atherosclerosis was detected in 20. 0% of women with PCOS compared with 3.3% of controls, supporting the presence of early carotid vascular injury in the PCOS group. These findings suggest that PCOS may be associated with measurable structural and hemodynamic vascular abnormalities before overt cardiovascular disease becomes clinically apparent [22]. However, interpretation should remain cautious because the study had a modest sample size and residual confounding by body mass index, insulin resistance, and hyperandrogenism could not be fully excluded.

Similarly, İncesu Çintesun et al. [21] demonstrated that mean carotid intima-media thickness was significantly higher in all PCOS phenotypes than in age-matched controls. When adjusted for body mass index, the greatest CIMT values were observed in phenotypes A and B, indicating that classic hyperandrogenic phenotypes may carry the greatest burden of subclinical vascular change. Although serum sclerostin concentrations were also higher in women with PCOS than in controls, no clear phenotype-specific relationship between sclerostin and atherosclerosis could be demonstrated. This study therefore provides strong imaging-based evidence of phenotype-related heterogeneity in vascular risk, while suggesting that sclerostin may be elevated in PCOS without functioning as a reliable marker of subclinical atherosclerosis across phenotypes [21]. Taken together, the direct vascular imaging studies provide the most clinically relevant evidence that PCOS may be associated with early vascular alterations in premenopausal women. However, these results should be interpreted with caution, given the number of imaging investigations was low and residual confounding could not be totally ruled out.

Taken together, the direct vascular imaging studies provide the most clinically relevant evidence that PCOS may be associated with early vascular alterations in premenopausal women. However, these results should be interpreted with caution, given the number of imaging investigations was low and residual confounding could not be totally ruled out.

In contrast, the adolescent study by Garoufi et al. [20] did not find a statistically significant difference in carotid intima-media thickness between adolescents with PCOS and healthy controls, nor between overweight/obese and healthy-weight PCOS subgroups. However, the absence of a structural cIMT difference should be interpreted alongside the study’s important metabolic findings. Overweight and obese adolescents with PCOS had significantly higher insulin levels, HOMA-IR, hsCRP, visceral adiposity index, and lipid accumulation product, as well as lower HDL-C, compared with healthy-weight adolescents with PCOS. In addition, cIMT correlated positively with systolic blood pressure and waist-to-height ratio. These results suggest that in adolescence, PCOS may be associated more consistently with an adverse metabolic and inflammatory profile than with already established structural vascular thickening, and that detectable vascular remodeling may emerge later in the disease course [20].

### 3.5. Indirect Biochemical and Dietary Surrogate Evidence

Two additional studies contributed indirect biochemical and dietary evidence rather than direct vascular imaging findings. In Bykowska-Derda et al. [23], all participants had PCOS and were stratified according to atherogenic index of plasma. Women in the high-AIP group were significantly less likely to consume low-glycaemic-index foods and selected whole-grain foods frequently than women in the low-AIP group. These findings support an association between less favorable dietary pattern and a more atherogenic biochemical profile in women with PCOS, but they do not constitute direct evidence of structural subclinical atherosclerosis [23]. Interpretation is further limited because the study lacked a non-PCOS comparator group and relied on dietary self-report and indirect biochemical surrogate markers rather than imaging-confirmed vascular outcomes.

Likewise, Fadhil et al. [24] found that women with PCOS had significantly higher serum podocalyxin, triglycerides, total cholesterol, LDL-C, VLDL-C, TC/HDL-C ratio, LDL/HDL-C ratio, and atherogenic index of plasma, alongside significantly lower HDL-C than healthy controls. Podocalyxin was positively correlated with AIP, suggesting possible early endothelial dysfunction and atherogenic alteration in women with PCOS. Nevertheless, because these findings were based on laboratory surrogate markers rather than imaging-confirmed vascular outcomes, they should be interpreted as supportive but indirect evidence of cardiovascular risk rather than definitive evidence of subclinical atherosclerosis [24]. In addition, the single-center case-control design and limited adjustment for obesity, insulin resistance, and other metabolic confounders reduce the certainty of causal interpretation.

Taken together, the clinical studies suggest that PCOS may be associated with early markers of subclinical vascular injury, particularly in adult women and in hyperandrogenic phenotypes, while the evidence in adolescents appears to favor early metabolic and inflammatory disturbance before overt structural vascular change becomes detectable. The most persuasive evidence comes from direct imaging studies, whereas biochemical and dietary studies provide indirect support for an unfavorable cardiometabolic milieu associated with PCOS [20,21,22,23,24].

The biochemical and dietary studies support the presence of an adverse atherogenic profile in women with PCOS, but they do not provide direct evidence of structural vascular disease. Elevated lipid ratios, atherogenic index of plasma, podocalyxin, and unfavorable dietary patterns may indicate increased cardiovascular risk, but these markers should be interpreted as indirect evidence. They should not be considered equivalent to imaging-confirmed subclinical atherosclerosis.

### 3.6. Exploratory Mechanistic and Bioinformatics Evidence

Four additional studies used transcriptomic, bioinformatics, and machine-learning approaches to investigate shared molecular mechanisms between PCOS and atherosclerosis. In Zhang et al. [26], 41 PCOS-related genes were identified in atherosclerosis, and MMP9 and P2RY13 emerged as hub genes with good diagnostic performance. Functional analyses indicated that inflammatory and immune-response pathways may represent central shared mechanisms [26].

In Wang et al., six hub genes—CD163, LAPTM5, TNFSF13B, MS4A4A, FGR, and IRF1—were identified, and pathway analyses again highlighted immune and inflammatory signaling as major shared biologic processes [27].

Similarly, Luo et al. identified DAPK1 as a key diagnostic biomarker linking PCOS and atherosclerosis, with findings suggesting involvement in T cell-mediated immune responses. The inclusion of validation datasets and cell experiments strengthened the biologic plausibility of this candidate marker [25].

Finally, Tang et al., identified 66 shared mitochondria-related differentially expressed genes, with GSTK1 emerging as the key shared mitochondria-related gene. Their results suggested that mitochondrial dysfunction and immune microenvironment changes may contribute to the overlap between PCOS and atherosclerosis [28].

Although these bioinformatics studies provide useful mechanistic context, they remain exploratory and hypothesis-generating. Because these studies were based primarily on transcriptomic databases and machine-learning models rather than clinical vascular assessment, they should not be interpreted as direct evidence of subclinical atherosclerosis in women with PCOS. They should not be interpreted as equivalent to direct clinical evidence of subclinical atherosclerosis, but rather as supportive mechanistic literature that may help explain why vascular abnormalities occur in some women with PCOS. Across these exploratory studies, a consistent theme was the involvement of immune-inflammatory signaling, oxidative stress-related pathways, and mitochondrial dysfunction, which may represent important directions for future translational research [25,26,27,28].

Bioinformatics and machine-learning studies provide mechanistic context by identifying inflammatory, immune, oxidative stress, and mitochondrial pathways that may link PCOS with atherosclerosis. However, these findings remain exploratory and hypothesis-generating. They do not show evidence of subclinical atherosclerosis in women with PCOS and hence should be viewed as exploratory mechanistic data rather than direct clinical vascular evidence.

### 3.7. Overall Synthesis of Findings

Overall, the included evidence indicates that the association between PCOS and subclinical atherosclerosis is strongest when assessed using direct vascular imaging in adult women, particularly among those with hyperandrogenic phenotypes or unfavorable metabolic profiles. Indirect biochemical and dietary studies support the presence of an atherogenic internal milieu in PCOS but do not by themselves confirm structural vascular disease. The exploratory bioinformatics literature further suggests that shared inflammatory, immune, and mitochondrial pathways may underline this association. However, the findings remain constrained by heterogeneity in study design, outcome definitions, age groups, and the relative balance between direct and surrogate evidence [20,21,22,23,24,25,26,27,28].

## 4. Discussion

This systematic review provides a clinically focused synthesis of the association between polycystic ovary syndrome (PCOS) and subclinical atherosclerosis by clearly distinguishing direct vascular evidence from indirect biochemical and mechanistic findings. The strongest evidence emerged from studies using vascular imaging, which demonstrated early arterial changes in premenopausal women with PCOS, particularly among those with hyperandrogenic phenotypes. However, the overall certainty of evidence remained low according to GRADE assessment because the available data were predominantly observational, heterogeneous, and based on relatively small study populations. In contrast, indirect biochemical and exploratory studies primarily supported the presence of an adverse cardiometabolic profile rather than definitive structural vascular disease [29,30].

A key contribution of the revised synthesis is the prioritization of direct imaging outcomes over indirect surrogates. In our review, the most persuasive evidence came from studies demonstrating increased carotid wall thickness and altered hemodynamics in adult women with PCOS, whereas the adolescent study did not show a clear difference in cIMT despite the presence of adverse metabolic abnormalities. This pattern aligns with recent reviews suggesting that vascular injury in PCOS is likely progressive and may become more apparent with age, duration of metabolic exposure, and accumulation of cardiometabolic risk factors. Contemporary reviews also note that young reproductive-age cohorts often have low absolute cardiovascular event rates, making subclinical vascular markers especially relevant in this population [31,32].

The phenotype-specific findings in our review are also noteworthy. The observation that hyperandrogenic phenotypes carried the highest imaging burden supports the view that PCOS should not be interpreted as a uniform cardiometabolic entity. Emerging literature suggests that cardiovascular risk in PCOS varies across phenotypic components and may be especially influenced by ovulatory dysfunction, menstrual irregularity, and hyperandrogenism, although the relative contribution of each component remains incompletely resolved. In particular, systematic phenotype-focused work has shown that oligo-amenorrhea is associated with increased cardiovascular morbidity independent of obesity, while the evidence for isolated hyperandrogenism is more mixed. This may help explain why the classical phenotypes in our synthesis appeared more metabolically and vascularly adverse than the non-hyperandrogenic phenotype [5,33].

The adolescent findings deserve separate consideration. In the younger cohort, cIMT did not differ significantly despite clear evidence of insulin resistance, inflammation, and adverse lipid-related risk markers in the overweight/obese subgroup. This divergence between structural vascular measures and metabolic abnormalities may indicate that the atherosclerotic process in PCOS begins with cardiometabolic dysregulation before a measurable arterial phenotype becomes detectable. Such an interpretation is supported by recent reviews emphasizing that obesity, visceral adiposity, insulin resistance, and dyslipidemia may precede overt vascular remodeling and may amplify cardiovascular vulnerability over time, particularly when present from adolescence onward [34,35].

The biochemical and dietary studies in our review provide supportive, but not confirmatory, evidence. Elevated AIP, unfavorable lipid ratios, and podocalyxin suggest an atherogenic and endothelial stress profile in women with PCOS, while reduced intake of low-glycaemic-index foods appears to cluster with worse lipid-associated risk [36]. However, these findings remain indirect because they do not directly establish arterial wall change. This distinction is important, especially recent reviews cautioning that many cardiovascular studies in PCOS rely on markers that sit along the pathway to atherosclerosis rather than measuring vascular disease itself. As a result, surrogate markers should be viewed as complementary to, rather than substitutes for, direct imaging outcomes [37,38].

The exploratory bioinformatics findings in our review should be interpreted in the same cautious framework. Although the identified genes differed across studies, the convergent signal around immune-inflammatory pathways, oxidative stress, and mitochondrial dysfunction is biologically plausible and consistent with contemporary mechanistic literature [39]. Recent work has highlighted endothelial dysfunction as a central link between the endocrine-metabolic features of PCOS and later cardiovascular risk. For example, vascular imbalance involving reduced nitric oxide bioavailability and disturbed vasodilatory–vasoconstrictive signaling has been documented in women with PCOS, while a recent meta-analysis reported significantly lower nitric oxide levels in PCOS compared with controls [40]. Together, these findings strengthen the plausibility that endothelial dysfunction is not simply an epiphenomenon, but an early stage in the cardiovascular phenotype of PCOS [41].

From a clinical perspective, the present findings support a stratified rather than uniform interpretation of cardiovascular risk in PCOS. The data do not justify concluding that all premenopausal women with PCOS already have subclinical atherosclerosis; however, they do support the view that women with hyperandrogenic phenotypes, insulin resistance, central adiposity, and adverse lipid or inflammatory profiles are more likely to exhibit early vascular abnormalities. This position is consistent with the 2023 international evidence-based PCOS guideline, which now emphasizes awareness of increased cardiovascular risk and recommends comprehensive risk assessment within routine care [42]. It is also consistent with recent cardiovascular reviews arguing that earlier attention to blood pressure, adiposity, glycemic status, menstrual irregularity, and lipid abnormalities may be more clinically useful than waiting for late cardiovascular outcomes to emerge [43].

At the same time, the review findings should be interpreted with caution. The available literature remains limited by small sample sizes, cross-sectional or case-control designs, incomplete confounder control, and substantial heterogeneity in age groups, outcomes, and diagnostic thresholds. Recent reviews have underscored these same limitations, noting that short follow-up duration, phenotype inconsistency, and variability in obesity adjustment remain major barriers to drawing firm causal conclusions about cardiovascular disease in PCOS. This is especially relevant in subclinical atherosclerosis research, where differences in imaging protocols and surrogate definitions can materially affect effect estimates and comparability across studies [44].

Future research should therefore move in three directions. First, larger prospective phenotype-stratified cohorts are needed to determine whether the vascular abnormalities detected in young and middle-aged women with PCOS translate into a sustained excess of atherosclerotic cardiovascular events across the life course. Second, direct vascular outcomes—such as carotid imaging, arterial stiffness, coronary calcium, or validated endothelial function measures—should be prioritized over isolated biochemical surrogates. Third, mechanistic studies should be integrated with well-characterized clinical phenotyping so that candidate pathways, including immune activation, nitric oxide dysregulation, and mitochondrial stress, can be linked more convincingly to patient-level vascular outcomes. This integrated approach is increasingly advocated in recent PCOS cardiovascular literature and would help bridge the gap between molecular plausibility and clinically actionable prediction.

The findings of this review have important implications for clinical practice, research, and preventive women’s health. First, the evidence suggests that PCOS should not be viewed solely as a reproductive disorder, but rather as a condition with potential early vascular and cardiometabolic consequences, particularly among women with hyperandrogenic phenotypes and unfavorable metabolic profiles. The strongest evidence in this review came from direct vascular imaging studies, which showed increased carotid wall thickness and altered hemodynamic parameters in adult women with. These findings support the need for greater cardiovascular awareness in the routine assessment of women with PCOS, especially in those with obesity, insulin resistance, dyslipidemia, or androgen excess.

Second, the findings highlight the value of phenotype-informed risk stratification. Not all women with PCOS appear to carry the same degree of vascular risk, and the evidence in this review suggests that classical hyperandrogenic phenotypes may be more strongly associated with subclinical atherosclerotic change than non-hyperandrogenic phenotypes. This has implications for individualized follow-up and early prevention strategies. In younger patients, particularly adolescents, the absence of measurable structural vascular change should not be interpreted as absence of risk, because adverse metabolic and inflammatory patterns may already be present and may precede detectable arterial remodeling.

Third, the indirect biochemical and dietary findings suggest that easily obtainable markers such as lipid ratios, atherogenic index of plasma, and endothelial-related biomarkers may help identify women with PCOS who warrant closer cardiometabolic surveillance, although these should not replace direct vascular assessment when structural vascular outcomes are the research or clinical target.

This review should be interpreted in light of several important limitations. First, the included evidence base was relatively small and methodologically heterogeneous. The clinical studies varied substantially in design, age group, sample size, comparator structure, and outcome definition, which limited direct comparability across studies and reduced the feasibility of drawing strong quantitative conclusions. In particular, only a subset of included studies used direct vascular imaging measures, whereas others relied on biochemical or dietary surrogate markers that are informative but indirect indicators of atherosclerotic risk.

Second, most of the clinical evidence came from observational studies, including cross-sectional and case–control designs, which preclude causal inference. Residual confounding remains a major concern, particularly with respect to adiposity, insulin resistance, androgen excess, dietary patterns, and other metabolic risk factors that may independently influence vascular outcomes. Third, the evidence from adolescents and adults may not be directly comparable, as vascular remodeling may emerge over time and may not yet be detectable in younger cohorts despite clear metabolic disturbance. This may partly explain the contrast between the negative structural findings in adolescents and the positive imaging findings in adult women.

Fourth, although bioinformatics and machine-learning studies were included to provide mechanistic context, these studies were based on public transcriptomic datasets and should not be interpreted as equivalent to patient-level clinical evidence. Their inclusion strengthens biologic plausibility but may also introduce conceptual heterogeneity if not clearly separated from the principal clinical synthesis. Finally, the review was limited to published English-language studies and was affected by restricted access to some full texts during the screening process, which may have introduced selection bias and reduced completeness of the evidence base.

## 5. Limitations

This systematic review has several limitations. First, a substantial number of potentially eligible full-text reports (32 of 50) could not be retrieved despite database access efforts, reference screening, and retrieval attempts, which may have introduced selection bias and reduced the completeness of the evidence base. Second, the review was restricted to studies published between 2021 and 2026 to capture contemporary evidence; however, this restriction may have excluded earlier high-quality imaging studies that could have contributed important foundational evidence regarding subclinical atherosclerosis in women with PCOS. Third, the included studies were heterogeneous with respect to PCOS diagnostic criteria, phenotype distribution, participant age, body mass index, vascular outcome definitions, and adjustment for potential confounding factors, limiting direct comparability across studies. Fourth, most included clinical studies were observational, and several were cross-sectional, precluding causal inference regarding the relationship between PCOS and subclinical atherosclerosis. Fifth, the number of studies providing direct vascular imaging evidence was relatively small, and several included modest sample sizes with limited adjustment for important metabolic confounders, including obesity, insulin resistance, dyslipidemia, and hyperandrogenism. Finally, although biochemical, transcriptomic, and bioinformatics studies were included to provide supportive mechanistic insights, they were considered exploratory and hypothesis-generating and should not be interpreted as equivalent to direct clinical evidence of subclinical atherosclerosis.

## 6. Conclusions

Current evidence supports a possible link between PCOS and early vascular changes in premenopausal women, especially in investigations using direct vascular imaging. But evidence is still scarce and varied. Larger prospective phenotype-stratified studies with standardised vascular evaluations are needed to clarify cardiovascular risk in this cohort.

## Figures and Tables

**Table 1:** PICOS framework for the review question.

PICOS Element	Description
Population (P)	Premenopausal or reproductive-age women with a confirmed diagnosis of polycystic ovary syndrome (PCOS) based on recognized diagnostic criteria, including the Rotterdam criteria, National Institutes of Health (NIH) criteria, or Androgen Excess Society (AES) criteria.
Exposure/Condition (I/E)	Presence of PCOS, regardless of disease duration, treatment status, body mass index, or phenotypic subtype.
Comparator (C)	Women without PCOS, including healthy controls or non-PCOS comparison groups matched or unmatched for age, body mass index, or other relevant baseline characteristics.
Primary Outcomes (O)	Direct markers of subclinical atherosclerosis and early vascular change, including carotid intima-media thickness (CIMT), carotid intima thickness, wall shear stress, arterial stiffness parameters, endothelial function, and other validated imaging- or vascular function-based measures.
Secondary Outcomes (O)	Validated biochemical surrogate markers associated with early atherosclerotic risk, including the atherogenic index of plasma (AIP), lipid-associated atherogenic markers, and endothelial dysfunction biomarkers where reported alongside the clinical context.
Exploratory Outcomes (O)	Mechanistic or hypothesis-generating findings, including transcriptomic, molecular, or bioinformatic markers, described separately and not considered equivalent to direct clinical vascular evidence.
Study Design (S)	Human observational studies, including cross-sectional, case–control, and cohort studies. Interventional studies were eligible only if baseline human vascular data relevant to the review question were reported.
Setting	No restriction by country or healthcare setting.
Time frame	Studies published between January 2021 and January 2026.
Language	English-language publications only.

**Table 2:** Database-specific search strategy and keywords used for study identification.

Database	Search Terms/Keywords Used *
PubMed/MEDLINE	(“Polycystic Ovary Syndrome”[Mesh] OR “PCOS”[tiab] OR “polycystic ovarian syndrome”[tiab] OR “polycystic ovary disease”[tiab] OR “hyperandrogenism”[tiab]) AND (“Atherosclerosis”[Mesh] OR “Carotid Intima-Media Thickness”[Mesh] OR “CIMT”[tiab] OR “subclinical atherosclerosis”[tiab] OR “endothelial dysfunction”[tiab] OR “arterial stiffness”[tiab] OR “wall shear stress”[tiab] OR “vascular imaging”[tiab]) AND (“premenopausal”[tiab] OR “reproductive age”[tiab] OR “women”[tiab] OR “female”[Mesh])
Embase	(‘polycystic ovary syndrome’/exp OR ‘PCOS’:ti,ab OR ‘polycystic ovarian syndrome’:ti,ab OR ‘hyperandrogenism’:ti,ab) AND (‘atherosclerosis’/exp OR ‘carotid intima media thickness’/exp OR ‘CIMT’:ti,ab OR ‘subclinical atherosclerosis’:ti,ab OR ‘endothelial dysfunction’:ti,ab OR ‘arterial stiffness’:ti,ab OR ‘wall shear stress’:ti,ab OR ‘vascular imaging’:ti,ab) AND (‘premenopausal’:ti,ab OR ‘reproductive age’:ti,ab OR ‘woman’/exp)
Scopus	TITLE-ABS-KEY (“polycystic ovary syndrome” OR PCOS OR “polycystic ovarian syndrome” OR hyperandrogenism) AND TITLE-ABS-KEY (atherosclerosis OR “carotid intima-media thickness” OR CIMT OR “subclinical atherosclerosis” OR “endothelial dysfunction” OR “arterial stiffness” OR “wall shear stress” OR “vascular imaging”) AND TITLE-ABS-KEY (premenopausal OR “reproductive age” OR women OR female)
Web of Science Core Collection	TS=(“polycystic ovary syndrome” OR PCOS OR “polycystic ovarian syndrome” OR hyperandrogenism) AND TS=(atherosclerosis OR “carotid intima-media thickness” OR CIMT OR “subclinical atherosclerosis” OR “endothelial dysfunction” OR “arterial stiffness” OR “wall shear stress” OR “vascular imaging”) AND TS=(premenopausal OR “reproductive age” OR women OR female)
Cochrane Library	(“polycystic ovary syndrome” OR PCOS OR “polycystic ovarian syndrome”):ti,ab,kw AND (atherosclerosis OR “carotid intima-media thickness” OR CIMT OR “subclinical atherosclerosis” OR “endothelial dysfunction” OR “arterial stiffness” OR “wall shear stress” OR “vascular imaging”):ti,ab,kw AND (premenopausal OR “reproductive age” OR women OR female):ti,ab,kw

Abbreviations: PCOS = polycystic ovary syndrome; CIMT = carotid intima-media thickness. * Search terms should be adapted to the syntax and indexing system of each database. Limits applied: English language; publication period January 2021 to January 2026.

**Table 3:** Risk of bias assessment of included human clinical studies.

Study	Study Design	Selection Bias	Exposure/Diagnosis Ascertainment	Outcome Measurement	Confounding	Other Concerns	Overall Risk of Bias
Garoufi et al. (2021) [20]	Cross-sectional	Moderate—small sample and adolescent-only recruitment may limit representativeness	Low—PCOS diagnosed using Rotterdam criteria with exclusion of major alternative diagnoses	Low to moderate—cIMT and laboratory markers were objectively measured, but single-time-point design limits robustness	Moderate to high—limited ability to control for obesity and metabolic confounders in a small cross-sectional sample	Restricted generalizability to adolescents; no longitudinal follow-up	Moderate
İncesu Çintesun et al. (2021) [21]	Prospective controlled study	Low to moderate—age-matched controls and clear exclusions, but single-center recruitment	Low—Rotterdam-based diagnosis with systematic phenotypic subgrouping	Low—CIMT and biochemical variables assessed objectively	Moderate—some adjustment reported, but residual confounding by metabolic factors remains possible	Single-center design; relatively limited external generalizability	Low to moderate
Dong et al. (2025) [22]	Prospective controlled study	Low to moderate—appropriate age-matched controls, though modest sample size	Low—clinically defined PCOS and comprehensive baseline assessment	Low—direct vascular assessment with UHFUS and vector flow imaging	Moderate—BMI, hyperandrogenism, and insulin resistance differed between groups and may partially confound observed associations	Modest sample size; no long-term outcome follow-up	Low to moderate
Bykowska-Derda et al. (2023) [23]	Cross-sectional	Moderate—PCOS-only sample without external control group	Low—Rotterdam criteria clearly applied	Moderate—AIP is an accepted biochemical surrogate, but not a direct vascular measure; diet was self-reported	Moderate to high—age and BMI considered in analyses, but residual dietary and metabolic confounding likely	Recall/reporting bias in dietary questionnaire; indirect outcome only	Moderate
Fadhil et al. (2023) [24]	Case–control	Moderate—single-center recruitment may limit representativeness	Low—Rotterdam criteria used with explicit exclusion criteria	Moderate—laboratory measurements were objective, but outcomes were surrogate biochemical markers rather than imaging-confirmed vascular change	Moderate to high—limited adjustment for metabolic and anthropometric confounders	Surrogate endpoints only; modest sample size	Moderate

**Table 4:** Data extraction summary of included studies examining PCOS and subclinical atherosclerosis-related outcomes.

A. Human clinical studies included in the principal evidence synthesis									
Study	Country	Study design	Sample and groups	PCOS diagnostic criteria	Age	Main outcome(s) assessed	Assessment method	Key findings	Evidence category
Garoufi et al. (2021) [20]	Greece	Cross-sectional study	32 adolescents with PCOS and healthy age-matched controls; within-PCOS subgroup analysis by weight status (23 overweight/obese; 9 healthy weight)	Rotterdam criteria	12.4–18.4 years; mean PCOS age approximately 16.3 years	cIMT; insulin resistance; inflammatory and lipid-related cardiovascular risk markers	Carotid intima-media thickness measurement plus clinical and laboratory cardiometabolic assessment	cIMT did not differ significantly between PCOS adolescents and controls or between overweight/obese and healthy-weight PCOS subgroups. However, overweight/obese adolescents with PCOS had significantly higher insulin, HOMA-IR, hsCRP, VAI, and LAP, and lower HDL-C. cIMT correlated positively with systolic blood pressure and waist-to-height ratio.	Direct vascular imaging evidence, but negative structural finding with supportive metabolic risk profile
İncesu Çintesun et al. (2021) [21]	Turkey	Prospective controlled study	134 women with PCOS and 33 age-matched controls; PCOS subgrouped into phenotype A (*n* = 35), B (*n* = 33), C (*n* = 31), and D (*n* = 35)	Rotterdam criteria	Adult premenopausal women	CIMT; serum sclerostin; hormonal and metabolic profile	Carotid intima-media thickness measurement and serum biomarker analysis	Mean CIMT was significantly higher in all PCOS phenotypes than in controls. BMI-adjusted CIMT was highest in phenotypes A and B. Serum sclerostin was higher in PCOS than in controls, but no clear association between sclerostin and atherosclerosis across phenotypes was demonstrated.	Strong direct vascular imaging evidence with phenotype-specific risk stratification
Dong et al. (2025) [22]	China	Prospective controlled study	40 women with PCOS and 30 age-matched healthy controls	Rotterdam criteria reported in manuscript synthesis; study recruited clinically defined PCOS women	Mean age 29 ± 5 years in PCOS group and 30 ± 6 years in controls	Intima thickness (IT); intima-media thickness (IMT); wall shear stress (WSS); subclinical atherosclerosis prevalence	Ultra-high-frequency ultrasound and vector flow imaging	Subclinical atherosclerosis was detected in 20.0% of women with PCOS versus 3.3% of controls. PCOS patients had significantly higher IT and IMT and lower WSS in the common carotid artery and carotid bulb. Findings supported early carotid vascular injury in PCOS.	Strongest direct imaging evidence of early vascular injury
Bykowska-Derda et al. (2023) [23]	Poland	Cross-sectional study	127 women with PCOS only; grouped into high AIP (>0.11) and low AIP (≤0.11)	Rotterdam criteria	Reproductive-age women, 18–40 years	Atherogenic index of plasma (AIP); dietary intake patterns; body composition	Biochemical lipid-based risk assessment; KomPAN dietary questionnaire; body composition assessment	Women in the high-AIP group were significantly less likely to consume low-glycaemic-index foods and selected whole-grain foods frequently. The study supports an association between dietary pattern and atherogenic risk in PCOS, but does not directly measure structural vascular change.	Indirect biochemical and dietary surrogate evidence
Fadhil et al. (2023) [24]	Iraq	Case–control study	63 women with PCOS and 61 healthy controls	Rotterdam 2003 criteria	18–38 years	Serum podocalyxin; lipid profile; AIP; lipid risk ratios	Serum biomarker assay and lipid profile analysis	PCOS women had significantly higher podocalyxin, triglycerides, total cholesterol, LDL-C, VLDL-C, TC/HDL-C ratio, LDL/HDL-C ratio, and AIP, with lower HDL-C than controls. Podocalyxin correlated positively with AIP, suggesting possible early endothelial and atherogenic alterations.	Indirect biochemical surrogate evidence
B. Exploratory bioinformatics and machine-learning studies retained as mechanistic evidence only									
Study	Country/data source	Study design	Data source/samples	Main analytic approach		Principal biomarker(s)/gene(s) identified	Main findings		Evidence category
Zhang et al. (2024) [26]	GEO datasets	Bioinformatics and machine learning study	PCOS dataset GSE54248 and atherosclerosis datasets GSE100927 and GSE28829	Differential expression analysis, WGCNA, PPI, LASSO, SVM-RFE, RF, ROC, nomogram, immune infiltration analysis		MMP9, P2RY13	Identified 41 PCOS-related genes in atherosclerosis. MMP9 and P2RY13 emerged as hub genes with good diagnostic performance, and findings suggested inflammatory and immune-response pathways as shared mechanisms.		Exploratory mechanistic evidence
Wang et al. (2025) [27]	GEO datasets	Bioinformatics and machine learning study	PCOS datasets GSE10946, GSE34526, GSE137684; atherosclerosis datasets GSE100927, GSE28829, GSE43292	DEG screening, WGCNA, PPI, LASSO, SVM, RF, ROC, nomogram, immune infiltration analysis		CD163, LAPTM5, TNFSF13B, MS4A4A, FGR, IRF1	Six hub genes were identified with strong diagnostic performance. Functional analyses highlighted shared immune and inflammatory pathways linking PCOS and atherosclerosis.		Exploratory mechanistic evidence
Luo et al. (2024) [25]	GEO datasets and validation experiments	Bioinformatics, WGCNA, machine learning, and validation study	Four PCOS datasets and two atherosclerosis datasets from GEO, with external validation and cell experiments	DEG analysis, WGCNA, machine learning, immune infiltration analysis, GSEA, validation datasets, cell experiments		DAPK1	DAPK1 was identified as a key diagnostic biomarker linking PCOS and atherosclerosis, with suggested involvement in T cell-mediated immune responses. Experimental validation supported its biological relevance.		Exploratory mechanistic evidence
Tang et al. (2026) [28]	GEO datasets and MitoCarta3.0	Bioinformatics and machine learning study	PCOS datasets GSE54250 and GSE114419; atherosclerosis datasets GSE28829, GSE43292, GSE100927; mitochondria-related genes from MitoCarta3.0	DEG screening, LASSO, enrichment analysis, immune infiltration analysis, validation cohorts		GSTK1	Identified 66 shared mitochondria-related differentially expressed genes. GSTK1 emerged as the key shared mitochondria-related gene and was associated with immune microenvironment changes in both conditions.		Exploratory mechanistic evidence

## Data Availability

No new data were created or analyzed in this study.

## Supplementary Materials

The following supporting information can be downloaded at https://www.mdpi.com/article/10.3390/jcm15135197/s1. PRISMA 2020 Checklist. Reference [45] is cited in the Supplementary Materials.

## References

[1] Yasmin A., Roychoudhury S., Sengupta P., Ahmed A.B.F., Ranjan Madhu N., Paul Choudhury A., Kolesarova A., Dutta S., Maldonado Rosas I. (2025). Prevalence of polycystic ovary syndrome (PCOS) and its associated hormonal and comorbid risk factors in Northeast India: A cross-sectional comparative study. Ther. Adv. Reprod. Health.

[2] Christ J.P., Cedars M.I. (2023). Current Guidelines for Diagnosing PCOS. Diagnostics.

[3] Singh S., Pal N., Shubham S., Sarma D.K., Verma V., Marotta F., Kumar M. (2023). Polycystic Ovary Syndrome: Etiology, Current Management, and Future Therapeutics. J. Clin. Med..

[4] Prosperi S., Chiarelli F. (2025). Insulin resistance, metabolic syndrome and polycystic ovaries: An intriguing conundrum. Front. Endocrinol..

[5] Geraci G., Riccio C., Oliva F., Gabrielli D., Colivicchi F., Grimaldi M., Facchinetti F., Unfer V. (2025). Women with PCOS have a heightened risk of cardiometabolic and cardiovascular diseases: Statement from the Experts Group on Inositol in Basic and Clinical Research and PCOS (EGOI-PCOS) and Italian Association of Hospital Cardiologists (ANMCO). Front. Cardiovasc. Med..

[6] Yang C.-W., Guo Y.-C., Li C.-I., Liu C.-S., Lin C.-H., Liu C.-H., Wang M.-C., Yang S.-Y., Li T.-C., Lin C.-C. (2020). Subclinical Atherosclerosis Markers of Carotid Intima-Media Thickness, Carotid Plaques, Carotid Stenosis, and Mortality in Community-Dwelling Adults. Int. J. Environ. Res. Public Health.

[7] Moreau K.L., Hildreth K.L. (2014). Vascular Aging across the Menopause Transition in Healthy Women. Adv. Vasc. Med..

[8] Kicińska A.M., Maksym R.B., Zabielska-Kaczorowska M.A., Stachowska A., Babińska A. (2023). Immunological and Metabolic Causes of Infertility in Polycystic Ovary Syndrome. Biomedicines.

[9] Palomba S., Santagni S., Falbo A., La Sala G.B. (2015). Complications and challenges associated with polycystic ovary syndrome: Current perspectives. Int. J. Womens Health.

[10] Gomez J.M.D., VanHise K., Stachenfeld N., Chan J.L., Merz N.B., Shufelt C. (2022). Subclinical cardiovascular disease and polycystic ovary syndrome. Fertil. Steril..

[11] Bizuneh A.D., Joham A.E., Teede H., Mousa A., Earnest A., Hawley J.M., Smith L., Azziz R., Arlt W., Tay C.T. (2025). Evaluating the diagnostic accuracy of androgen measurement in polycystic ovary syndrome: A systematic review and diagnostic meta-analysis to inform evidence-based guidelines. Hum. Reprod. Update.

[12] Eghleilib M., Eghlileb M., Eghlileb A. (2026). Cardiovascular Outcomes Associated With Biologic Therapy in Inflammatory Dermatologic Diseases: A Systematic Review. Cureus.

[13] Herman R., Trsan J., Lipar L., Jensterle M., Janez A. (2025). Endocrine Adaptations to Prolonged Fasting: From Physiology, Clinical Uncertainties, Translational Challenges to Healthspan Implications. Nutrients.

[14] Alruwaili A.N., Alruwaili M.M., Ramadan O.M.E., Ali S.I., Shaban M. (2024). Nursing strategies for enhancing calm in older Arabs with dementia: Integrating Snoezelen methods, aromatherapy, and personal items to reduce agitation. Geriatr. Nurs..

[15] Mohamed S.A.A.K., Shaban M. (2024). Age and expertise: The effects of ageism on professional recognition for senior nurses. Geriatr. Nurs..

[16] Nashwan A.J., Abou Hashish E.A., Mohamed A.S., Alrimawi I., Aqtam I., Al Obeisat S., Alhalaiqa F., Alzaatreh M., Al Hadidi M., AL-Fayyadh S. (2024). Exploring the National Nursing Research Priorities in the Eastern Mediterranean Region and Overcoming the Associated Challenges: An Expert Opinion. Cureus.

[17] Yuan H., Zhou J., Li H., Kang S., Park S. (2025). A Systems Biology Approach to Memory Health: Integrating Network Pharmacology, Gut Microbiota, and Multi-Omics for Health Functional Foods. Int. J. Mol. Sci..

[18] Osibogun O., Ogunmoroti O., Kolade O.B., Hays A.G., Okunrintemi V., Minhas A.S., Gulati M., Michos E.D. (2022). A Systematic Review and Meta-Analysis of the Association Between Polycystic Ovary Syndrome and Coronary Artery Calcification. J. Womens Health.

[19] Sena C.M., Gonçalves L., Seiça R. (2022). Methods to evaluate vascular function: A crucial approach towards predictive, preventive, and personalised medicine. EPMA J..

[20] Garoufi A., Pagoni A., Papadaki M., Marmarinos A., Karapostolakis G., Michala L., Soldatou A. (2022). Cardiovascular Risk Factors and Subclinical Atherosclerosis in Greek Adolescents with Polycystic Ovary Syndrome: Its Relationship with Body Mass Index. Children.

[21] İncesu Çintesun F.N., Can Ü., Çintesun E., Altunkeser A., Kaya A., Günenç O. (2021). Serum sclerostin level and its relation to subclinical atherosclerosis in the polycystic ovary syndrome phenotypes: A prospective controlled study. Turk. J. Obstet. Gynecol..

[22] Dong Y., Hong S., Wang Z., Song D., Liu M., Zeng J., Du Y., Xu J., Shi L., Gao W. (2025). Evaluation of subclinical atherosclerosis in women with polycystic ovary syndrome based on ultra-high-frequency ultrasound and vector flow imaging. Quant. Imaging Med. Surg..

[23] Bykowska-Derda A., Kałużna M., Garbacz A., Ziemnicka K., Ruchała M., Czlapka-Matyasik M. (2023). Intake of Low Glycaemic Index Foods but Not Probiotics Is Associated with Atherosclerosis Risk in Women with Polycystic Ovary Syndrome. Life.

[24] Fadhil N.M., Hamdi R.A., Abdulhameed K.M., Jaber L.A. (2023). Assessment of the risk of subclinical atherosclerosis in women with polycystic ovary syndrome. Bionatura.

[25] Luo Y., Zhou Y., Jiang H., Zhu Q., Lv Q., Zhang X., Gu R., Yan B., Wei L., Zhu Y. (2024). Identification of potential diagnostic genes for atherosclerosis in women with polycystic ovary syndrome. Sci. Rep..

[26] Zhang W., Wu Y., Yuan Y., Wang L., Yu B., Li X., Yao Z., Liang B. (2024). Identification of key biomarkers for predicting atherosclerosis progression in polycystic ovary syndrome via bioinformatics analysis and machine learning. Comput. Biol. Med..

[27] Wang L., Zhang Y., Ji F., Si Z., Liu C., Wu X., Wang C., Chang H. (2025). Identification of crucial genes for polycystic ovary syndrome and atherosclerosis through comprehensive bioinformatics analysis and machine learning. Int. J. Gynecol. Obstet..

[28] Tang L., Long W., Chen Q., Chen J., Zhang Y., Nie Z., Li W. (2026). Key mitochondria-related genes and molecular mechanisms shared between polycystic ovary syndrome and atherosclerosis: A bioinformatics and machine learning study. Medicine.

[29] Teede H.J., Mousa A., Tay C.T., Costello M.F., Brennan L., Norman R.J., Pena A.S., Boyle J.A., Joham A., Berry L. (2024). Summary of the 2023 international evidence-based guideline for the assessment and management of polycystic ovary syndrome: An Australian perspective. Med. J. Aust..

[30] Lee I., Dokras A., Alur-Gupta S. (2025). Association of polycystic ovary syndrome with atherosclerotic cardiovascular disease events. Fertil. Steril..

[31] Pililis S., Lampsas S., Kountouri A., Pliouta L., Korakas E., Livadas S., Thymis J., Peppa M., Kalantaridou S., Oikonomou E. (2024). The Cardiometabolic Risk in Women with Polycystic Ovarian Syndrome (PCOS): From Pathophysiology to Diagnosis and Treatment. Medicina.

[32] Profili N.I., Castelli R., Gidaro A., Manetti R., Maioli M., Petrillo M., Capobianco G., Delitala A.P. (2024). Possible Effect of Polycystic Ovary Syndrome (PCOS) on Cardiovascular Disease (CVD): An Update. J. Clin. Med..

[33] Lo A.C.Q., Lo C.C.W., Oliver-Williams C. (2023). Cardiovascular disease risk in women with hyperandrogenism, oligomenorrhea/menstrual irregularity or polycystic ovaries (components of polycystic ovary syndrome): A systematic review and meta-analysis. Eur. Heart J. Open.

[34] Karoli R., Fatima J., Siddiqi Z., Vatsal P., Sultania A.R., Maini S. (2012). Study of early atherosclerotic markers in women with polycystic ovary syndrome. Indian J. Endocrinol. Metab..

[35] Wu X., Wilke M., Batara J., Proctor S., Cree M., Ghosh M., Raggi P., Windram J., Becher H., Vine D. (2025). Atherogenic ApoB-dyslipidemia, atherosclerotic cardiovascular disease, cardiac dysfunction and remodeling in high-risk young women with and without polycystic ovary syndrome: A pilot study. Front. Endocrinol..

[36] Demirci H., Menekse B., Ucgul E., Onaran Y., Bayram S.M., Cakal E. (2025). Impact of polycystic ovary syndrome on the atherogenic plasma index: A retrospective analysis. BMC Endocr. Disord..

[37] Çolak A., Özpelit M.E., Okyay R.E., Kumral Z., Özpelit E. (2023). Cardiac Functions and Peripheral Arterial Stiffness in Patients with Polycystic Ovary Syndrome: A Cross-Sectional Study. Cyprus J. Med. Sci..

[38] Pourahmad B., Majidnia M., Saniee N., Riyahifar S., Moradi Y. (2025). Polycystic ovary syndrome with stroke, hypertension, and cardiovascular diseases: A systematic review and meta-analysis. BMC Womens Health.

[39] Liu S., Liu J., Wang Y., Deng F., Deng Z. (2025). Oxidative Stress: Signaling Pathways, Biological Functions, and Disease. MedComm.

[40] Kıran T.R., Erdem M., Yıldırım E., İnceoğlu F. (2025). Vascular imbalance in polycystic ovary syndrome: Insights into endothelial dysfunction. PLoS ONE.

[41] Hosseini S., Shabani F., Nayebzadeh M., Asadi F., Kabiranaraki Y., Asadpour M., Golavar Y., Asadi S., Etezadi A. (2026). Neutrophils and NETosis in polycystic ovary syndrome: Unraveling the immuno-metabolic thromboinflammatory axis. Eur. J. Med. Res..

[42] Teede H.J., Tay C.T., Laven J.J.E., Dokras A., Moran L.J., Piltonen T.T., Costello M.F., Boivin J., Redman L.M., Boyle J.A. (2023). Recommendations From the 2023 International Evidence-based Guideline for the Assessment and Management of Polycystic Ovary Syndrome. J. Clin. Endocrinol. Metab..

[43] Gilani A., Umar K., Gilani F., Ahmad M., Abbasi M.S., Yaseen M., Zeeshan M., Ullah N., Waseem A., Batool F. (2024). The Effect of Glycemic Control on Cardiovascular Disease Progression in Adults With Early-Onset Type 2 Diabetes: A Longitudinal Cohort Analysis. Cureus.

[44] Garg P.K., Bhatia H.S., Allen T.S., Grainger T., Pouncey A.L., Dichek D., Virmani R., Golledge J., Allison M.A., Powell J.T. (2024). Assessment of Subclinical Atherosclerosis in Asymptomatic People In Vivo: Measurements Suitable for Biomarker and Mendelian Randomization Studies. Arterioscler. Thromb. Vasc. Biol..

[45] Page M.J., McKenzie J.E., Bossuyt P.M., Boutron I., Hoffmann T.C., Mulrow C.D., Shamseer L., Tetzlaff J.M., Akl E.A., Brennan S.E. (2021). The PRISMA 2020 statement: An updated guideline for reporting systematic reviews. BMJ.

